# Allogeneic anorectal transplantation in rats: technical considerations and preliminary results

**DOI:** 10.1038/srep30894

**Published:** 2016-08-04

**Authors:** Flavio H. F. Galvão, Daniel R. Waisberg, Victor E. Seid, Anderson C. L. Costa, Eleazar Chaib, Rachel Rossini Baptista, Vera Luiza Capelozzi, Cinthia Lanchotte, Ruy J. Cruz, Jun Araki, Luiz Carneiro D’Albuquerque

**Affiliations:** 1 Laboratory of Experimental Transplant Surgery, LIM-37, Department of Gastroenterology, Faculdade de Medicina da Universidade de São Paulo (FMUSP), São Paulo, Brazil; 2 Department of Pathology FMUSP, São Paulo, Brazil; 3 Intestinal Rehabilitation and Transplant Center, University of Pittsburgh Medical Center, Pittsburgh, PA, USA; 4 Department of Plastic Surgery, University of Tokyo, Tokyo, Japan

## Abstract

Fecal incontinence is a challenging condition with numerous available treatment modalities. Success rates vary across these modalities, and permanent colostomy is often indicated when they fail. For these cases, a novel potential therapeutic strategy is anorectal transplantation (ATx). We performed four isogeneic (Lewis-to-Lewis) and seven allogeneic (Wistar-to-Lewis) ATx procedures. The anorectum was retrieved with a vascular pedicle containing the aorta in continuity with the inferior mesenteric artery and portal vein in continuity with the inferior mesenteric vein. In the recipient, the native anorectal segment was removed and the graft was transplanted by end-to-side aorta-aorta and porto-cava anastomoses and end-to-end colorectal anastomosis. Recipients were sacrificed at the experimental endpoint on postoperative day 30. Surviving animals resumed normal body weight gain and clinical performance within 5 days of surgery. Isografts and 42.9% of allografts achieved normal clinical evolution up to the experimental endpoint. In 57.1% of allografts, signs of immunological rejection (abdominal distention, diarrhea, and anal mucosa inflammation) were observed three weeks after transplantation. Histology revealed moderate to severe rejection in allografts and no signs of rejection in isografts. We describe a feasible model of ATx in rats, which may allow further physiological and immunologic studies.

Fecal incontinence, often accompanied by definitive colostomy, is one of the most psychologically and socially debilitating medical conditions. The incidence of fecal incontinence in the general population may be as high as 17%, and the number of patients undergoing colostomy for this reason is steadily increasing^1^. Various techniques for anal reconstruction have been reported to minimize the deleterious effects of a definitive colostomy, including sphincteroplasty, gracilis or gluteus maximus muscle transfer, and artificial sphincter implantation. However, none of these procedures is considered a gold standard therapy for patients requiring anorectal reconstruction^2^. Recently, allotransplantations of non-vital organs, such as the face^3^, larynx^4^, extremities^5^,^6^, and uterus^7^ have been successfully performed to improve patients’ quality of life. In the emerging field of regenerative medicine, this new transplantation modality, called vascularized composite allotransplantation (VCA), is currently the ultimate resource for patients suffering from highly complex surgical problems. Thus, we can speculate about the benefit of the addition of an anorectal segment through intestinal/colonic allografts for individuals with severe fecal incontinence and permanent colostomy, especially in candidates for intestinal or multivisceral transplantation.

Anorectal transplantations (ATx) have been described experimentally in rat^8^,^9^,^10^,^11^, pig^12^,^13^, dog^14^,^15^, and human cadaver^16^ models. Most of these studies evaluated the technical aspects of autologous transplantation, whereas others covered complex and hardly reproducible surgical techniques. In this study, we have developed an innovative microsurgical model of allogeneic anorectal transplantation in rats. We herein describe in detail the technical aspects of this model, and evaluate the clinical outcomes and histological changes of ATx within the first month post-transplant.

## Materials and Methods

### Animals

Twenty-two male rats weighing 220–280 grams were included in this study. Four isogeneic (Lewis-to-Lewis) and seven allogeneic (Wistar-to-Lewis) isolated ATx procedures were performed. All procedures followed the guidelines of the International Council for Laboratory Animal Science and received approval from the University of São Paulo Medical School ethical committee. Fasting was imposed on all rats for 24 h prior to the operation, but they were given drinking water *ad libitum*. Every effort was made to minimize animal suffering, including the administration of pain medication and antibiotics.

### Donor operation

**Figure 1:**
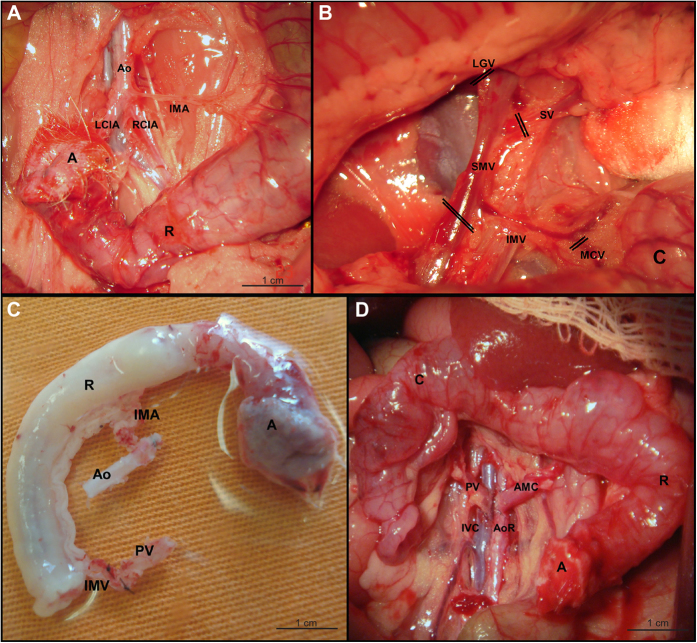
Surgical procedures of anorectal transplantation. (**A**) Anorectal segment mobilized to the inside of the abdominal wall, preserving the inferior mesenteric artery (IMA). (**A)** anus; Ao, abdominal aorta; LCIA and RCIA, left and right common iliac artery, respectively; R, rectum. (**B**) Superior mesenteric vein (SMV) and portal tributaries divided during graft removal. The inferior mesenteric vein (IMV) is preserved. (**C**) colon; LGV, left gastric vein; MCV, middle colic vein; SV, splenic vein. (**C**) Graft containing the anorectal segment with a vascular pedicle including the abdominal aorta (Ao) in continuity with the inferior mesenteric artery (IMA) and the portal vein (PV) in continuity with the inferior mesenteric vein (IMV) to enhance the vessels diameter to simplify the anastomosis. (**A**) anus; R, rectum. (**D**) Graft macroscopic aspect following reperfusion. (**A**) anus; AMC, aortomesenteric conduit; AoR, recipient’s abdominal aorta; (**C**) colon; IVC, inferior vena cava; PV, portal vein; R, rectum.

Four Lewis and seven Wistar rats were used as isogeneic and allogeneic donors, respectively. After intaperitoneal anesthesia with ketamine (30 mg/kg) and xylazine (10 ml/kg), we performed a midline laparotomy and perianal incision to procure the anorectal graft. The perineal dissection was performed adjacent to the rectal segment, and the pudendal nerves were not preserved. The anorectal segment was then mobilized into the abdomen through the perineum, preserving the inferior mesenteric artery (IMA) (Fig. 1A). A long segment of the aorta, from the renal arteries to its bifurcation, was isolated in order to prepare an aortic conduit (graft inflow). The branch from the joint of the inferior mesenteric vein (IMV) and middle colonic vein was isolated. The middle colic, splenic, and left gastric veins were then divided (Fig. 1A). Next, 1500 UI of heparin was injected through the penile vein. The superior mesenteric vein (SMV) and the portal vein (PV) were separated from the pancreas. The SMV was dissected up to the PV next to the liver and was tied before IMV drainage (Fig. 1B). The PV was transected near the hepatic hilum. A long venous segment containing the mesenteric vessels in continuity with the PV was carefully manipulated (graft outflow). The aorta distal to the IMA was tied and transected. The proximal part of the abdominal aorta adjacent to the renal vessels was also transected (aortic conduit).

The graft was then removed from the abdominal cavity. The aorta was then cannulated and flushed with 20 mL of cold lactated Ringer’s solution. The colon and rectum were also flushed with the same cold solution. The composite graft was placed in a container with 50 mL of cold lactated Ringer’s solution and stored at 4 °C (Fig. 1C).

### Recipient operation

Eleven Lewis rats were used as both isogeneic and allogeneic recipients. The recipients received ceftriaxone (50 mg/kg, intramuscularly) and metronidazole (7.5 mg/kg, intravenously). After the same anesthesia used for the donors, a combined perineal and abdominal incision, the anorectal segment was mobilized and resected as described above. Vascular reconstruction was started by an end-to-side aortic-aortic anastomosis followed by an end-to-side porto-cava anastomosis with a 10-0 nylon running suture (Fig. 1D). All microvascular anastomoses were performed by the same surgeon (FHFG). After reperfusion, digestive tract continuity was restored. After colostomy, the anal segment was placed into its original position and attached to the perineum. We carried out daily administration of ceftriaxone (50 mg/kg, intramuscularly) for three days after surgery.

### Postoperative assessment

Clinical examination was performed by assessing surgical complications (abdominal distention, lethargic posture, and diarrhea), behavioral modifications, anal aspect, body weight, and survival. Body weight loss of more than 30%, declining clinical status, and one-month animal survival were set as experimental endpoints. Samples from the proximal graft border and anal canal (including anal sphincters and the surrounding skin) were removed at the end of the study for histological examination using hematoxylin-eosin. Classification of intestinal graft rejection was graded as indeterminate, mild, moderate, and severe, as described in Table 1. This score system is based in the previously classic scoring grade described by Wu *et al*. for intestinal allograft rejection^17^ and includes specific changes in the three segments of anorectal graft (rectum, anal canal and perianal skin).

## Results

A trained microsurgeon performed the microsurgical technique and the whole procedure was completed in approximately 90 min. (donor: 29 ± 19 min. and recipient: 57 ± 38 min.). The mean of time consuming for end-to-side porto-cava anastomosis in recipient was 15.6 ± 3.1 min. and the entire vascular anastomosis time was 27.8 ± 5.7 min.

One animal that underwent isogeneic transplantation died 5 days after transplant due to technical failure (9.1%). The cause of death was graft necrosis associated with peritonitis due to thrombosis of the PV.

**Figure 2:**
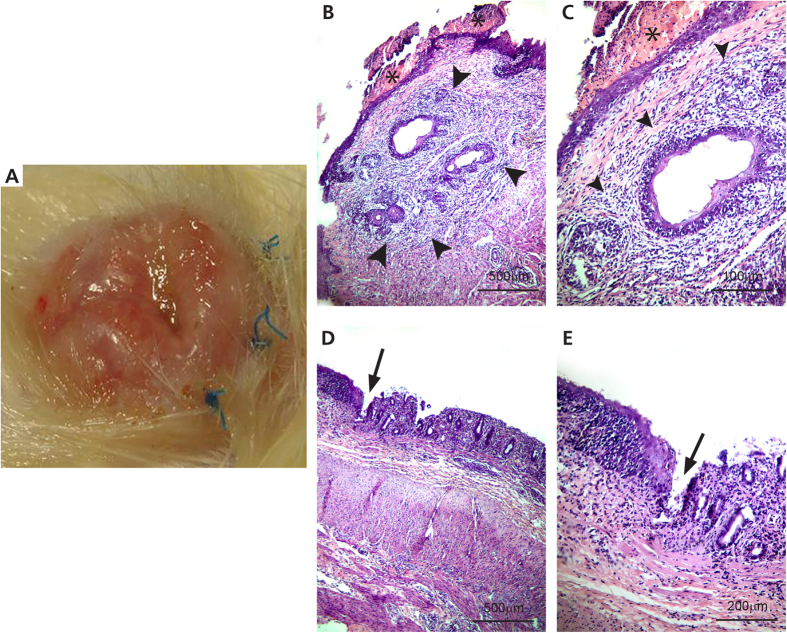
Postoperative immunological rejection of allogeneic anorectal tansplantation. (**A**) Macroscopic aspect of the anus 21 days after allotransplantation showing hyperemia and signs of rejection. (**B**,**C)** Histopathology of the anus showing lymphocytic infiltration in both the internal and the external anal sphincters. (**D**,**E**) Histopathology of the rectum showing severe rejection with villous damage in the superficial mucosa and considerable amounts of lymphocytic cryptitis, vasculitis, and necrosis.

All surviving animals sustained mild body weight loss (<10%) within the first week and progressively regained normal body weight. Stool characteristics returned to normal by the end of the first week in most of the animals (90.1%). Six animals (three isogeneic and three allogeneic transplants) retained favorable evolution until the study end-point. Four animals receiving allogeneic transplants (57.1% of allografts) presented mild clinical signs of rejection approximately 20 days after transplant, including diarrhea (n = 1), abdominal distention (n = 3), and anal hyperemia and petechiaes (n = 3) (Fig. 2A). The clinical outcomes and histological classification of all eleven animals are summarized in Table 2.

All animals were sacrificed at postoperative day 30. In every animal receiving isogeneic transplants, macroscopic examination showed a small amount of abdominal adhesions and normal graft appearance. Histopathology confirmed normal graft appearance without signs of rejection. On the other hand, in most animals receiving allogeneic transplants, a significant amount of intestinal adhesions, diffuse graft inflammation, and enlarged Peyer’s patches could be observed. Three moderate and four severe graft rejections were identified according to the criteria^18^. Histological analysis of the allografts sustaining severe rejection showed lymphocytic inflammatory infiltration throughout the intestinal wall (Fig. 2B). Albeit diffuse, inflammatory infiltrate was predominant in the lamina propria. Mucosal ulcerations, atrophic epithelium, and gland rarefaction were also noted. The submucosae presented diffuse edema, with dissociated collagen fibers and extensive lymphocytic infiltrate surrounding small caliber vessels. Muscular layers exhibited diffuse lymphocytic infiltrate and edema, with few necrotic muscular fibers. The main feature of anal canal rejection was perianal skin with predominant subepidermic lymphocytic inflammatory infiltrate permeated by edema and apoptotic cells. There was also marked atrophy in the squamous epithelium and diffuse lymphocytic infiltrate in both internal and external anal sphincters with diffuse muscular edema (Fig. 2C).

## Discussion

In this study, we described an innovative and feasible microsurgical model for isolated ATx in rats. We also showed for the first time the different immunological responses and rejection patterns between isogeneic and allogeneic ATx. In 2000, O’Bichere *et al*.^12^ described for the first time an experimental model of ATx. They performed four transplants in swine focusing on the technical aspects and feasibility of the procedure. However, the abovementioned study had several limitations, including short follow-up time (24 h), high cost, lack of immunosuppression, and prolonged operation time (mean of 372 min.). Therefore, this study presents some advantages including low cost, shorter operation time (approx. 90 min.), and the requirement of only a single microsurgeon to perform the whole procedure.

In this article we portrait the first experience of ATx using arterial and venous microanastomosis. This technique was inspired by our previous intestinal transplantation models in the rat^17^,^18^,^19^ and may allow studies about this transplanted segment toward graft function and immunological reactions including rejection and tolerance induction.

In our previous ATx experiments in rodents we compared a simulated model of autologous ATx with animals submitted to anorectal segment resection and colostomy^8^. In the simulated ATx group, the animals achieved a good post-operative evolution with restoration of normal defecation status and body weight gain within the first week of post-transplant. On the other hand, animals without anorectal segment and colostomy achieved miserable clinical evolution with intense diarrhea, important body weight loss and high mortality rate (i.e. the group). Recently, Seid *et al*.^11^, using a refined simulated autologous ATx model, observed good post-operative evolution of the transplanted animals and total functional recovery of the transplanted anorectal segment after two weeks assessed by manometry.

The disadvantage of these autologous ATx models includes a simulated transplantation technique and the impossibility to study the graft rejection. In the present model we performed the real transplantation of the graft by recovering it from one rat and reimplanted in another using vascular anastomosis. Furthermore, in the current model we can perform syngeneic transplantations (between identical rat strains) to study graft functional recovery and preservation surveys, and allogeneic transplantations (between different rat’s strain) to perform investigations about rejection and tolerance induction.

Araki *et al*.^9^ described a notable anal autotransplantation model where the anal canal was implanted by supermicrosurgery of the vascular pedicles including the bilateral internal pudendal artery and vein, along with the pudendal nerve. Even though this technique is interesting, it enclose the drawback of requiring a difficult supermicrosurgery for the vascular anastomosis, since the pudendal vessels diameters are extremely small (<0.4 mm). Furthermore, the authors did not describe the evolution of the transplanted rats.

Tanabe *et al*. investigated the evolution of small bowel transplantation by different donor–recipient allograft combinations in 1994^20^. They observed mortality of all rats due to severe graft rejection between 5 and 14 days after surgery. In the current model, no death due to graft rejection occurred up to postoperative day 30. Furthermore, we observed that the Wistar-to-Lewis allogeneic combination of rats was less aggressive than that reported in small bowel allograft rejection. Late rejection has also been observed in models of colon transplantation^21^. The tolerogenic aspect of this segment remains an open issue for exploration in further experiments.

The used four histologic features for score grading in this investigation based in Wu *et al*. system are useful and relatively easily to perform because identify steady features of acute cellular rejection in the three segments of the graft (rectum, anal canal and skin). This features include crypt apoptosis, crypt epithelial injury, activated lymphocytic inflammatory infiltration in the lamina propria (rectum), epithelium and gland atrophy (canal anal), epidermic and subepidermic activated lymphocytic inflammatory infiltrate, squamous epithelium atrophy (skin), arterites, necrosis, architectural distortion and ulceration. These features were similar in the three segments of anorectal graft analyzed for each degree of rejection. The activated lymphocytes mixed with some eosinophils and neutrophils in the rejection of ATx are easily differentiated from other nonspecific conditions of lymphocytes infiltration that contain nonactivated lymphocytes. The intensity of the infiltration was generally correlated with the severity of the rejection.

Recently Zutshi *et al*. have shown that anal resting pressure in the rat is maintained by both the external and internal anal sphincters (EAS and IAS, respectively)^23^. Continuous pressure waves are observed even after pudendal nerve transection, indicating that most of the contraction pressure comes from the smooth muscle. These findings allowed these authors to speculate that the IAS is the major contributor to anal resting pressure in rats. In another study from the same group, Salcedo *et al*. showed that rats were not rendered incontinent after bilateral pudendal nerve transection^24^. In fact, differences in resting pressure and electromyography amplitude and frequency were not statistically significant from controls in the long-term. Interestingly, the authors also observed that both the EAS and IAS recovered over time after sphincterotomy in rats. In our model, the pudendal nerves were not preserved during graft removal nor reconstructed during recipient surgery. Nevertheless, we observed normal stool aspect one week after transplantation. This observation suggests that the intrinsic rectal innervation and regenerative capacity of anal sphincters might be sufficient for the recovery of anorectal function in rats, even with complete disconnection of the innervation during transplantation.

The dissection of the extraperitoneal rectum must be near the rectum serosa to avoid urinary complications and injury to the iliac vessels. The IMA and IMV were dissected far from its wall to avoid spasm and thrombosis due to their manipulation. Another important consideration was the creation of long aortic-mesenteric and porto-mesenteric conduits so the anus could be implanted in the perineum without tension on microvascular anastomoses. As observed by O’Bichere *et al*.^12^ and in our previous studies^8^, the arterial supply from the IMA was sufficient for adequate revascularization and regeneration of the graft. The extraperitoneal perineal conduct seems to provide sufficient vascularization for the distal rectum and anus.

In conclusion, we described a feasible model of ATx in rats that could be useful for further anorectal physiological and immunological studies. Ultimately, the current study may increase interest not only in ATx alone but also as a combined modality therapy with intestinal and multivisceral allografts in human clinical practice.

## Additional Information

**How to cite this article**: Galvão, F. H. F. *et al*. Allogeneic anorectal transplantation in rats: technical considerations and preliminary results. *Sci. Rep.* **6**, 30894; doi: 10.1038/srep30894 (2016).

## Figures and Tables

**Table 1:** Histologic principles for classifying anorectal allograft acute rejection.

Grade	Histologic Findings
Indeterminate for ACR	Rectum–Mucosa, submucosa and muscular layers with minimum lymphocytic infiltration and edema and no crypt epithelial injury and no ulceration. Increased crypt cell apoptosis, but with less than six apoptotic bodies per 10 crypts.Anal channel–Minimum lymphocytic infiltrate and edema in mucosa, submucosa and both internal and external anal sphincters.Perianal skin–Minimum subepidermic lymphocytic infiltrate.
Mild rejection	Rectum–Altered mucosae architecture (e.g. mild villi blunting) with minor activated lymphocytic inflammatory infiltration predominantly in the lamina propria, rare crypt epithelial injury and no ulceration, submucosa and muscular layers with slight activated lymphocytic infiltrate and edema. Increased crypt cell apoptosis (>6 six apoptotic bodies/10 crypts).Anal channel–Incipient activated lymphocytic infiltrate and edema in mucosa, submucosa and both internal and external anal sphincters.Perianal skin–mild subepidermic lymphocytic inflammatory infiltrate.
Moderate rejection	Rectum–Mucosa, submucosa and muscular layers revealing disseminated activated lymphocytic infiltration, edema and increased crypt epithelial injury and apoptosis. Increased crypt cell apoptosis (>6 six apoptotic bodies/10 crypts), accompanied by foci of confluent apoptosis.Anal channel–Mucosa submucosa and both internal and external anal sphincters with intense lymphocytic infiltrate and diffuse edema, important epithelium and gland atrophy and sites of necrosis, arterites and ulceration.Perianal skin–Severe epidermic and subepidermic activated lymphocytic inflammatory infiltrate, moderate squamous epithelium atrophy architectural distortion. All grafts segments showed places of moderate architectural alteration.
Severe rejection	Rectum–Mucosa, submucosa and muscular layers exhibiting widely disseminated activated lymphocytic infiltration, intense crypt epithelial injury, diffuse apoptosis, important edema, transmural necrosis and ulceration.Anal channel–Mucosa submucosa and both internal and external anal sphincters with intense activated lymphocytic infiltrate and edema, important epithelium and gland atrophy and sites of necrosis.Perianal skin–Severe epidermic and subepidermic lymphocytic infiltrate, marked atrophy in the squamous epithelium. All grafts segments showed important and diffuse architectural distortion.

ACR–Acute cellular rejection.

**Table 2:** Model of acute celular rejection grade in anorectal allograft.

Type of the Transplant	Weight loss >10%	Complication	POD	Histological rejection grade
Isogeneic transplant (*L-L*)	no	none	—	None
	no	none	—	None
	no	none	—	None
	yes	Death 5^th^ POD	—	—
Allogeneic transplant *(W-L)*	no	none	—	Moderate
	no	none	—	Moderate
	no	none	—	Moderate
	no	Diarrhea, Abd distension	18^th^	Severe
	no	Abd distention, Anal hyperemia	21^st^	Severe
	no	Abd distention, Anal hyperemia	22^nd^	Severe
	no	Anal hyperemia	27^th^	Severe

Recipient characteristics, clinical evolution, and histological findings of 11 rats that underwent microsurgical isogeneic or allogeneic anorectal transplantation. *Abbreviations:* POD = postoperative day; W = Wistar; L = Lewis.
